# Molecular survey and interaction of common respiratory pathogens in chicken flocks (field perspective)

**DOI:** 10.14202/vetworld.2019.1975-1986

**Published:** 2019-12-16

**Authors:** Adel M. Abdelaziz, Mahmoud H. A. Mohamed, Mahmoud M. Fayez, Theeb Al-Marri, Ibrahim Qasim, Abdul Aziz Al-Amer

**Affiliations:** 1 Veterinary Educational Hospital, Faculty of Veterinary Medicine, Zagazig University, Egypt; 2 Department of Avian Diseases, Al Ahsa Veterinary Diagnostic Laboratory, Ministry of Environment, Water and Agriculture, Saudi Arabia; 3 Department of Clinical Sciences, College of Veterinary Medicine, King Faisal University, Saudi Arabia; 4 Department of Avian and Rabbit Medicine, Faculty of Veterinary Medicine, Zagazig University, Egypt; 5 Al Ahsa Veterinary Diagnostic Lab, Ministry of Environment, Water and Agriculture, Saudi Arabia; 6 Serum and Vaccine Research Institute, Abbassia, Egypt; 7 Department of Animal Resources, Ministry of Environment, Water and Agriculture, Riyadh, Saudi Arabia

**Keywords:** bidirectional interaction, chickens, molecular detection, respiratory pathogens

## Abstract

**Aim:**

The present study was designed for the detection of the most prevalent respiratory infections in chicken flocks and clarifying their interaction and impact on flock health.

**Materials and Methods:**

A total of 359 serum samples were collected from 55 backyard chickens and tested using commercial enzyme-linked immunosorbent assay kits to determine the seroprevalence of Newcastle disease virus (NDV), infectious bronchitis virus (IBV), influenza type A, *Mycoplasma gallisepticum* (MG), and *Mycoplasma synoviae* (MS). Molecular prevalence of NDV, IBV, low pathogenic avian influenza virus (LPAIV) H9N2, MG, and MS was carried out on swab, and tissue samples collected from 55 backyard flocks and 11 commercial broiler flocks suffered from respiratory infections using polymerase chain reaction (PCR) and reverse transcription-PCR.

**Results:**

Seroprevalence of NDV, IBV, Influenza type A virus, MG, and MS in chicken backyard flocks was 56.4%, 50.9%, 12.7%, 14.5%, and 3.6%, respectively. Specific antibodies against one or more respiratory viruses and mycoplasma were detected in 36.4% of backyard flocks, indicating concurrent viral infections. The molecular survey showed that 90.9% of chicken backyard flocks were infected with common respiratory viruses (NDV, IBV, and LPAIV H9N2) while 81.8% of commercial broiler flocks were infected. The molecular prevalence rate of NDV, IBV, and LPAIV H9N2 was 46.97%, 56.1%, and 19.7% in backyard flocks, respectively. Combined viral and bacterial infection represented 40% and 63.6% of the respiratory infections, resulting in enhanced pathogenicity and increased mortalities of up to 87.5% and 27.8% in backyard and commercial flocks, respectively. Mixed infection of IBV, LPAIV H9N2, and/or *Escherichia coli* is the most prevalent mixed infection in broiler flocks, inducing severe clinical outcomes. Avian pathogenic *E. coli* was, respectively, isolated from 40% of backyard flocks and 81.82% of broiler flocks. *Staphylococcus aureus* was isolated from three backyard chicken flocks mixed with other respiratory pathogens with elevated mortality. Mixed infection of *E. coli* and MG reported in 9.1% of broiler flock. MG was detected in 14.5% of backyard flocks and 9.1% of broiler flocks while MS was detected only in 3.6% of backyard chickens mixed with *E. coli*, and other viruses.

**Conclusion:**

Our results confirm that mixed infections are more commonly prevalent and associated with dramatic exacerbation in clinical outcomes than a single infection. Bidirectional synergistic interaction between these concurrently interacted respiratory pathogens explains the severe clinical impact and high mortality rate. The high prevalence of IBV (either as a single or combined infection) with LPAIV H9N2 and/or *E. coli*, in spite of intensive use of commercial vaccines, increases the need for revising vaccination programs and the application of standard biosecurity measures. Backyard chickens impose a great risk and threaten commercial flocks due to the high prevalence of viral respiratory pathogens.

## Introduction

The incidence and severity of respiratory infections in backyard and commercial broiler flocks due to viral infection and complicated pathogenic bacteria and mycoplasma increased recently in Saudi Arabia due to its intense poultry industry and vigorous changes in climatic conditions. Respiratory tract infections are of great importance in the poultry industry due to high mortality rates (MRs), high losses of body weight, and expensive use of medications, all of which influence net incomes. The etiology of respiratory affections is more complicated and has a multifactorial nature, often including more than one pathogen in the same flock, including infectious bronchitis virus (IBV), Newcastle disease virus (NDV), low pathogenic avian influenza virus (LPAIV) H9N2, *Escherichia coli, Staphylococcus aureus, Mycoplasma gallisepticum* (MG), and *Mycoplasma synoviae* (MS), these pathogens can cause respiratory diseases independently, or concurrently [1-3]. NDV caused by avian paramyxovirus 1, causes severe economic losses in the poultry industry. More than 200 avian species can be infected by various NDV strains [4,5]. The most severely affected species by NDV strains are chickens, turkeys, pheasant, and other gallinaceous species [4,6]. IBV is one of the most common respiratory affections and causes 100% morbidity with 25-80% mortality in chicks. The virus replicates in epithelial cells of the upper respiratory tract, producing different respiratory troubles, noisy respirations, and the formation of caseated plugs in tracheal bifurcation. Other IBV serotypes replicate mainly in epithelial cells of the kidney tubules or oviduct causing nephritis and decreased egg production [7-10]. Avian influenza virus (AIV) is members of the family *Orthomyxoviridae*. LPAIV H9N2 causes mild respiratory infection and a slight drop in egg production with MR <5% [11,12]. AIV of H9N2 subtype has been endemic in poultry in Asia and the Middle East [13,14]. It is clear that IBV infection maximized the pathogenicity and extended the period of H9N2 AIV shedding in chickens [15,16], increasing MR and economic losses, possibly due to mixed infection, and interaction with other respiratory pathogens [2,12,15-17].

Different studies showed that many organisms such as *S. aureus, Haemophilus paragallinarum, E. coli, Ornithobacterium rhinotracheale*, MG, MS, IBV, NDV, and even live IBV and NDV vaccines have synergistic effects that enhance the virulence of H9N2 and increase mortality in infected birds [7,18-23]. Such synergistic effects may be occurring through enhancing hyaluronic acid (HA) cleavage by secretion of trypsin-like proteases [18,24,25]. Viral infection may encourage concurrent bacterial infection by various mechanisms [26-29]. *E. coli* infection before, after, or concurrently with H9N2 virus infection could exacerbate the adverse effects of the H9N2 virus. *E. coli* and H9N2 together can mutually exacerbate the condition of either disease as compared to single infected birds [15,30].

Few research works described the extent of respiratory affections in chicken flocks in the Kingdom of Saudi Arabia [31,32]. Our study is considered the firs­t in this context, reporting a complete enumeration of the common respiratory pathogen and its interaction and synergistic effects that exacerbate its pathogenicity, leading to high economic losses, and mortalities in spite of vaccination programs.

In this study, we screened respiratory infected chicken flocks for the most common respiratory pathogens to state the interaction between different respiratory pathogens and their impact on chicken flocks, with the support of the Deputy Ministership for Animal Resources at the Ministry of Environment, Water and Agriculture (MEWA) of Saudi Arabia.

## Materials and Methods

### Ethical approval

All animal experiments were conducted according to the Animal Ethics protocols of the National Committee of Bio-Ethics, King Abdul-Aziz City of Science and Technology, Royal Decree No. M/59.

### Sampling

**Figure-1:**
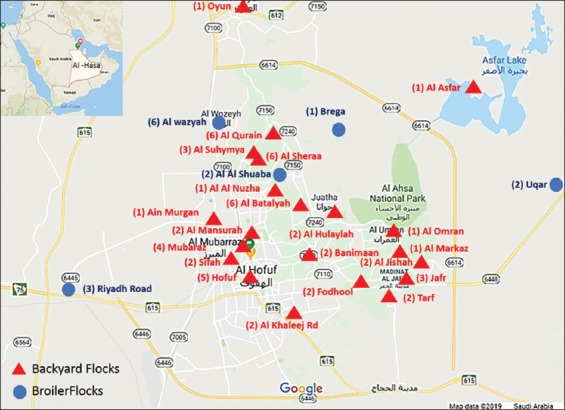
A map showing the distribution of sampled flocks in Al-Ahsa Province [Source: Map was designed by the authors with the help of Google Maps Tools].

From 2015 to 2017, 2857 samples were collected from 55 backyard chicken flocks, and 11 commercial broiler flocks, in 31 different localities in Al-Ahsa Eastern Region, Saudi Arabia (Figure-1). Examined flocks showed respiratory manifestation as dyspnea, sneezing, rales, sinusitis, eye lesions, and in some cases nervous sings with mortality for at least 3-7 days as investigated by the Department of Avian Diseases, Al-Ahsa Veterinary Diagnostic Laboratory, MEWA of Saudi Arabia. The samples consisted of serum (n=359), tracheal swabs (TS), and cloacal swabs (CS) (n=1242), and internal organs (n=1256); including trachea, lungs, liver, spleen, kidney, and brain after necropsy. Samples from each flock were pooled and treated separately. Blood samples were collected from brachial vein, and the collected sera were assessed for specific antibodies against the NDV, IBV, influenza A virus, MG, and MS by enzyme-linked immunosorbent assay (ELISA) test. Concurrently, TS, CS, and internal organs were taken from the morbid and necropsied birds and processed by molecular technique (polymerase chain reaction [PCR] and reverse transcription-PCR [RT-PCR]) (Tables-1 and 2).

**Table-1:** Details of examined backyard chicken flocks.

Flock		Sample size			MR	Cl. sings		PM	Flock		Sample size			MR	Cl. sings		PM
							
ID	Size	Se	Sw	Ti	%	Rs	Ns		ID	Size	Se	Sw	Ti	%	Rs	Ns
603	50	5	8	8	40	+	+	Fibrinous pericarditis and petich on provent	639	50	6	12	18	28	+	−	Tracheitis, nephrosis, general congestion
626	1000	16	28	32	15	+	−	Tracheal caseation	689	25	5	10	12	16	+	−	Airsacculitis, general congestion
704	300	5	6	-	2.3	**+**	−	-	693	300	5	10	15	15	+	+	Petch. on provent. and cecal tonsils
799	300	5	10	7	23.3	+	−	Congestion in lung, kidney, and ovary	739	80	5	10	15	37.5	+	−	Tracheitis, airsacculitis
826	100	5	10	4	29	+	+	Tracheitis and congestion of lung and kidney	771	80	7	14	20	87.5	+	+	Hemorrhagic laryngitis, tracheitis
874	45	7	10	20	46.7	**+**	−	Tracheitis and general congestion	796	1000	18	30	20	20	+	+	Petich. on provent. And cecal tonsils.
850	54	9	18	21	55.6	+	−	Laryngitis, tracheitis, ulcers on cecal tonsils	836	600	8	18	20	25	+	−	Yellow tracheal exudate and pluges
862	300	7	14	20	16.7	+	+	Laryngitis, tracheitis, and petich. on provent.	880	200	15	30	30	40	+	+	Hemorrhagic laryngitis, tracheitis
864	66	6	10	14	15.2	+	−	Petich. on provent and cecal tonsils,	1196	400	5	8	8	12.5	+	−	Tracheal exudate, general congestion
869	45	7	15	16	22.2	+	−	General congestion and petich. on coronary fat	1297	90	5	10	10	24.4	+	−	Tracheitis, airsacculitis and nephrosis
872	110	6	8	16	18.2	+	−	Petich. on provent., cecal tonsils	153	50	5	9	9	20	+	+	Petich. on provent., and cecal tonsils
930	800	5	10	12	8.8	+	−	Fibrinous pericarditis and airsacculitis	599	31	5	8	8	32.3	+	−	Tracheal exudate, petch. on provent
963	110	5	10	9	21.8	+	−	Tracheitis and airsacculitis	609	350	5	10	10	12.8	+	+	Petich. on provent. and cecal tonsils
1040	70	5	10	15	71.4	+	−	Tracheal exudate, nephrosis	37	50	5	10	10	18	+	−	Tracheal exudate
1058	150	5	6	6	33.3	+	−	General congestion	35	110	5	12	12	21.8	+	+	Petich. on provent. and cecal tonsils
1126	100	6	12	16	35	+	−	Tracheal exudate and organ congestion	101	80	7	14	12	16.3	+	−	Tracheitis and tracheal exudate
281	70	8	16	24	14.3	+	−	Tracheitis and pneumonia and fib. pericarditis	102	70	10	18	20	14.3	+	−	Tracheitis and tracheal exudate
325	1000	7	14	22	20	+	−	Fibrinous pericarditis and airsacculitis	103	120	5	10	11	19.2	+	−	Petich. on provent. and cecal tonsils
334	25	6	12	17	44	+	−	Petich. on provent. and cecal tonsils,	104	350	6	10	10	15.7	+	+	Petich. on provent. and cecal tonsils
385	300	7	16	18	30	+	−	Petich. on provent. and cecal tonsils	105	225	5	10	10	16.4	+	+	Petich. on provent. and cecal tonsils
322	30	4	8	6	33.3	+	+	Petich. on provent. and cecal tonsils	106	135	6	12	12	23	+	+	Petich. on provent. and cecal tonsils
320	80	4	8	6	6.25	+	−	Conjunctivitis, tracheitis and congestion	432	66	6	12	12	15.2	+	−	Tracheitis and tracheal exudate
384	800	6	12	18	27.3	+	+	Airsacculitis, and petch. on provent	451	92	6	10	10	23.9	+	+	Petich. on provent. and cecal tonsils
515	100	5	10	8	5	+	−	Tracheitis and airsacculitis	459	60	6	10	10	45	+	+	Petich. on provent. and cecal tonsils
551	10	5	10	10	60	+	−	Tracheal exudate, general congestion	978	1000	7	7	-	0.3	+	−	Tracheal congestion, general congestion
623	150	12	24	24	46.7	+	+	Laryngitis, tracheitis, and petch. on provent	1279	90	8	15	15	24.4	+	−	Airsacculitis, general congestion
653	200	5	10	10	25	+	+	Laryngitis, tracheitis, and general congestion	358	120	5	10	10	25	+	+	Pneumonia and petich. on provent
636	80	5	8	8	37.5	+	+	Laryngitis, tracheitis, and petch. on provent									

Cl. sings=Clinical sings, Rs=Respiratory sings, Ns=Nervous sings, Se=Serum, Sw=Swab, Ti=Tissue, MR=Mortality rate. Provent.=Proventriculus, Petch.= Petechial hemorrhages, PM=Postmortem

**Table-2:** Details of examined commercial broiler flocks.

Flock ID	Flock size/age^≠^	Vaccination	Sample size		MR %	Clinical sings		PM
	
			Swab	Tissues		Rs	Ns	
707	70000/8	ND+IB (1, 12 and 18 day)	30	42	0.4	+	−	General congestion, and omphalitis
950	525000/22	ND+IB (1, 12 day)	60	60	27.8	+	+	Tracheal pluges, airsacculitis, nephrosis, fibrinous pericarditis
971	25020/14	ND+IB (1, 12 day)	60	60	11.3	+	+	Tracheal pluges, airsacculitis, nephrosis, thymus congestion
1009	26066/15	ND+IB (1, 12 day)	50	80	12.4	+	+	Tracheal pluges, airsacculitis, nephrosis, thymus congestion
1054	10000/24	ND+IB (1, 12 day)	20	28	1.2	+	+	Congestion, fibrinous perihepatitis, and pericarditis. arthritis
1102	210000/19	ND+IB (1, 11 and 18 day)	60	40	12.9	+	+	Tracheal pluges, airsacculitis, nephrosis
1121	19400/26	ND+IB (1, 11 and 18 day)	60	50	8	+	−	Tracheal pluges, airsacculitis, nephrosis
1135	15000/18	ND+IB (1, 12 day)	50	40	13.3	+	−	Tracheal exudate, general congestion
502	21000/13	ND+IB (1, 12 day)	60	40	18	+	−	Pneumonia, fibrinous pericarditis, perihepatitis, splenomegaly
427	22500/21	ND+IB (1, 12 day)	60	40	12	+	−	Congestion in lung, kidney, fibrinous pericarditis
901	521000/17	ND+IB (1, 12 day)	60	40	27%	+	−	Tracheal caseation and pluges, fibrinous pericarditis

Ns=Nervous sings, Rs=Respiratory sings, MR=Mortality rate, ≠=Age/day. ND=Newcastle disease, IB=Infectious bronchitis, AI=Avian influenza, MG=*Mycoplasma gallisepticum*, MS=*Mycoplasma synoviae,* PM=Postmortem

### Serological survey

The specific antibodies against NDV, IBV, influenza type A, MG, and MS were detected using commercial ELISA kits (IDEXX, USA), as mentioned by the producer.

### Molecular screening

#### Detection of common respiratory viruses

Viral RNAs were extracted using MagNA Pure Compact Nucleic Acid Isolation Kit (Roche Diagnostics GmbH, Mannheim, Germany), according to the producer instruction. Extracted viral RNAs were stored at −80°C until used in subsequent molecular techniques.

One-step RT-PCR was carried out using an RT-PCR kit Qiagen. The primers employed in the RT-PCR (Table-3) were described in the previous studies for amplification of a 535 bp fragment of the fusion protein (F) gene of NDV [33], a 244 bp fragment of the M2 gene of AIV [34], a 430 bp fragment of the N gene of IBV [35], a 549 bp fragment of the H9 gene of AIV [36], and a 1420 bp fragment of the AIV N2 gene [32].

**Table-3:** Oligonucleotide primers for IBV, NDV, AIV, MG, and MS.

Pathogen	Primer name		Sequence	Annealing temperature (°C)	References
IBV	N primer430 bp	F Primer (N+) R Primer (N−)	5`-GAAGAAAACCAGTCCCAGATGCTTGG-3` 5`-GTTGGAATAGTGCGCTTGCAATACCG-3`	60	[35]
NDV	Primer F (APMV1-F) 535 bp	(APMV1-F-F) (APMV1-F-R)	5`-ATGGGCYCCAGACYCTTCTAC-3` 5`-CCTGAGGAGAGGCATTTGCTA-3`	60	[33]
AIV	AIV M2 detection 244 bp	M52C-M2-F 253R-M2-R	5/- CTTCTAACCGAGGTCGAAACG -3/ 5/- AGGGCATTTTGGACAAAGCGTCTA -3/	52	[34]
	AIV H9 typing 549 bp	H9-For H9-Rev	5/-ATT CAA GAC GCC CAA TAC AC-3/ 5/-TGA CCA ACC TCC CTC TAT GA-3/	52	[36]
	AIV N2 typing 1420 bp	AIVN2-F AIVN2-F	5/- GTAAAAATGAATCCAAATCAAAAG-3/ 5/- GTAAAAATGAATCCAAATCAAAAG-3/	52	[32]
MG	(mgC2) 237 bp	(F primer) (R primer)	5`-CGC AAT TTG GTC CTA ATC CCC AAC A -3` 5`-TAA ACC CAC CTC CAG CTT TAT TTC C-3`	72	[38]
MS	16S rRNA 214 bp	F primer R primer	5`-GAG AAG CAA AAT AGT GAT ATC A-3` 5`- CAG TGG TCT CCG AAG TTA ACA A-3`	72	[39]

NDV=Newcastle disease virus, IBV=Infectious bronchitis virus, MG=*Mycoplasma gallisepticum*, MS=*Mycoplasma synoviae*, AIV=Avian influenza virus

#### Detection of MG and MS

DNA was extracted from swab samples suspended in 1 ml of PCR-grade phosphate-buffered saline. The suspensions were centrifuged for 30 min at 14,000 g at 4°C. The supernatant was carefully removed, and the pellets were suspended in 25 μL PCR-grade water. The tube and the contents were boiled for 10 min and then placed on ice for 10 min before centrifugation at 14,000 g for 5 min. The supernatant was used as DNA temples for PCR reactions [37]. Extracted DNA was used in MG and MS PCR using the PCR Master Mix (Hot-start Taq PCR Master Mix Kit Qiagen) or stored at –70°C for later use. The primers employed in the PCR (Table-3) were described in the previous studies for amplification of a 237 bp fragment of the second cytoadhesin-like protein-encoding gene (mgc^2^) of MG [38], and a 214 bp fragment of 16S rRNA gene of MS [39] [32-36,38,39].

### Isolation and identification of respiratory pathogenic bacteria

Each sample was streaked on to 5% sheep blood agar, MacConkey, and brain heart infusion agar (Oxoid) and incubated aerobically at 37°C for 24 h. Isolates were permissively identified based on colony morphology, Gram staining, catalases, and oxidase tests [40]. Biochemical identification was carried out using VITEK 2 Compact (BioMérieux, France). The analysis was performed and interpreted according to the producer’s recommendations.

## Results

### Clinical findings and MRs in examined chicken flocks

Fifty-five backyard chicken flocks and 11 commercial broiler flocks from 31 different localities suffering from respiratory manifestation were examined clinically, postmortem (PM) findings were recorded, and samples were collected for laboratory investigations. Clinical manifestations and necropsy findings in respiratory infected flocks differed according to the causative agent and the type of infection, either single or mixed infections. The main observed clinical manifestations varied from mild to severe respiratory distress such as nasal/eye discharges, conjunctivitis, cough, gasping, rales, and difficult noisy respirations. In some flocks, respiratory signs conjoined with nervous manifestations as paresis, paralysis head shaking, opisthosomas, and circling. The main pathological lesions reported during PM examination were laryngeal/tracheal/lung congestion, tracheitis, tracheal discharges, congested visceral organs, airsacculitis, and fibrinous pericarditis, typical pathognomonic PM findings of VNDV included brain congestion, petechial hemorrhages on proventriculus, ulcerated intestinal mucosa, and cecal tonsils. Tracheal/lung congestion, as well as tracheal caseation at tracheal bifurcation that produced tracheal plugs besides kidney lesions in case of IBV infection, was recorded. More severe lesions were observed in the case of mixed infection with other viruses and bacteria. In the case of single infection with LPAIV (H9N2), mild respiratory manifestations were observed, while in mixed infection with other viruses and pathogenic bacteria, severe respiratory signs and lesions were observed with high MR (Tables-1 and 2).

The incidence of respiratory troubles increased in winter and spring.

### MR

Mixed infection of common respiratory viruses, pathogenic bacteria, and/or MG, and MS leads to the magnification of MRs, increasing economic losses. MR in non-vaccinated backyard flocks infected with NDV ranged from 12.8% to 44%. MR in backyard flocks suffering from a concurrent infection of NDV and *E. coli* ranged from 15% to 87.5%, while MR in backyard flocks has mixed NDV and IBV infection ranged from 25% to 71.4%. MR in flocks suffering from IBV infection ranged from 12.5% to 18%. In broiler flocks concurrently infected with IBV, H9N2, and *E. coli*, MR increased up to 27.8% (Table-2). In unvaccinated backyard flocks the mortality rate reached up to 71.4% and 60 % in flocks concurrently infected with IBV and NDV or IBV, H9N2 and *S. aureu* s respectively (Table-1).

LPAIV (H9N2) single infection was recorded in one backyard flock suffering from decreased feed intake, mild respiratory singes, conjunctivitis, and eye discharge with low MR (6.25%). Mixed LPAV (H9N2) infection with other viruses and bacteria was recorded in seven unvaccinated backyard flocks and six broiler flocks with enhanced pathogenicity, leading to more severe respiratory signs, and destructive lesions in respiratory organs and kidneys. MR was higher in backyard flocks coinfected with H9N2 and NDV (40%), H9N2, IBV, and *S. aureus* (60%), and H9N2, IBV, and MG (55.6%). On the other hand, MR in vaccinated broiler flocks coinfected with IBV and LPAIV (H9N2) ranged from 13.3% to 27% (Tables-1 and 2).

### Serological assay

Three hundred fifty-nine serum samples from 55 non-vaccinated backyard chicken flocks with respiratory infections were examined for antibodies to NDV, IBV, AI (type A), MG, and MS, while vaccinated broiler flocks were not investigated serologically.

The serological profile of the examined backyard chicken flocks revealed that the prevalence of NDV in backyard chickens was 56.4%, of which 34.5% were positive for NDV antibodies only, and 21.8% flocks had specific antibodies for NDV, IBV, and AIV (type A). IBV specific antibodies were detected in 50.9% of backyard chickens, of which 18.2% had IBV specific antibodies and 32.7% had mixed antibodies to NDV, AIV (type A), MG, and MS. AIV (type A) antibodies were detected in 12.7% of backyard flocks. Single AIV (type A) specific antibodies were detected serologically in only one flock (1.82%), while mixed antibodies for AIV (type A) with other pathogens such as NDV (3.6%), IBV (3.6%), IBV mixed with MG (1.82%), and IB mixed with MS (1.82%) were detected using ELISA tests. Specific antibodies for more than one virus and mycoplasma were detected in 36.4% backyard flocks (Table-4).

**Table-4:** Results of serological detection of respiratory pathogens in chicken backyard flocks.

Pathogen	Single infection					Mixed infection							Total (%)
	
	ND	IB	AI	MG	MS	ND+IB	ND+AI	ND+MG	IB+AI	IB+MG	IB+AI+MG	IB+AI+MS	
Total ND	19	-	0		-	10	2	0	0	0	0	-	31/55 (56.4)
Total IB	-	10	0	-	-	10	-	-	2	4	1	1	28/55 (50.9)
Total AI	-	-	1	-	-	-	2	0	2	-	1	1	7/55 (12.7)
Total MG	-	-	-	3	-	-		-	-	4	1	-	8/55 (14.5)
Total MS	-	-	-	-	1	-	-	0	-	-	-	1	2/55 (3.6)
Percent %	19	10	1	3	1	10	2	0	2	4	1	1	54/55 (98.2)

ND=Newcastle disease, IB=Infectious bronchitis, AI=Avian influenza, MG=*Mycoplasma gallisepticum*, MS=*Mycoplasma synoviae*

Serologically, the prevalence of MG and MS in backyard chicken flocks was 14.5% and 3.6%, respectively, mixed with common respiratory virus and other pathogenic bacteria (Table-4).

### Molecular assay

In backyard chicken flocks, 50 (90.9%) of the examined flocks showing respiratory manifestation were infected with common respiratory viruses (NDV, IBV, and LPAIV H9N2). The prevalence of NDV, IBV, and LPAIV (H9N2) infection was 56.36%, 50.9%, and 12.7%, respectively. Mixed infection of NDV and IBV was recorded in 10 flocks (18.2%), while LPAIV (H9N2) was detected in 7 (12.7%) flocks. Mixed infections of pathogenic bacteria and common respiratory viruses (NDV, IBV, and LPAV H9N2) were recorded in 29 flocks (40%). The prevalence of MG and MS was 14.5% and 3.6%, respectively. Mixed infections of common respiratory viruses and MG represent 9.1% of respiratory troubles in backyard chicken flocks (Tables-5).

**Table-5:** Results of molecular detection and bacterial isolation of respiratory pathogens in backyard flocks.

Pathogen	Single infection			Mixed viral infection			Mixed viral and bacterial infection										Single bacterial infection		Mixed bacterial infection		Total
					
	ND	IB	AI	ND +IB	IB +AI	ND +AI	ND +*E. coli*	ND+IB +*E. coli*	IB +*E. coli*	IB+AI +*E. coli*	IB+MG +*E. coli*	IB+AI +MG	IB+AI +MS	IB+AI +*Staphylococcus*	Total		*E. coli*	*Staphylococcus*	MG+ *E. coli*	MS+ *E. coli*	
Total ND																**Total viral infected flocks**					
yard	14	-	-	5	-	2	5	5	-	-	-	-		-			-	-	-	-	31/55 (56.36%)
bro.	-	-	-	-	-	-	-	-	-	-	-	-		-			-	-	-	-	0
Total IB																					
yard	-	6	-	5	-	-	-	5	4	-	4	1	1	2			-	-		-	28/55 (50.9%)
bro.	-	-	-	-	2	-	-	-	3	4	-	-	-	-			-	-		-	9/11 (81.82%)
Total AI																					
yard.	-	-	1	-	-	2	-	-	-	-	-	1	1	2			-	-	-	-	7/55 (12.7%)
bro.	-	-	-	-	2	-	-	-	-	4	-	-	-	-			-	-	-	-	6/11 (54.5%)
Total MG																					
yard.	-	-	-	-	-	-	-	-	-	-	4	1	-	-			-	-	3	-	8/55 (14.5 %)
bro.	-	-	-	-	-	-	-	-	-	-	-	-	-	-			-	-	1	-	1/11 (9.1%)
Total MS																					
yard.	-	-	-	-	-	-	-	-	-	-	-	-	1	-			-	-	-	1	2/55 (3.6%)
bro.	-	-	-	-	-	-	-	-	-	-	-	-	-	-			-	-	-	-	0
Total *E. coli*																					
yard.	-	-	-	-	-	-	5	5	4	-	4	-		-			-	-	3	1	22/55 (40%)
bro.	-	-	-	-	-	-	-	-	3	4	-	-	-	-			-	-	1	-	9/11 (81.82%)
Total *Staphylococcus*																					
yard.	-	-	-	-	-	-	-	-	-	-	-	-		2			-	1	-		3/55 (5.5%)
bro.	-	-	-	-	-	-	-	-	-	-	-	-	-	-			-	-	-	-	0
Total/(%)																				
yard.	14 25.5%	6 10.9%	1 1.82%	5	0	2	5	5	4	0	4	1	1	2	22/55 40%	50/55 90.9%	0	1	3	1	55/55
bro.	0	0	0	0	2	0	0	0	3	4	0	0	0	0	7/11 63.6%	9/11 81.8%	1/11	0	1/11	0	11/11

yard=Backyard flocks, bro.=Broiler flocks. ND=Newcastle disease, IB=Infectious bronchitis, AI=Avian influenza, MG=*Mycoplasma gallisepticum*, MS=*Mycoplasma synoviae*, *E. coli*=*Escherichia coli*

Pathogenic *E. coli*, respiratory viruses (NDV and IBV), and mycoplasma were detected concurrently in 40% of examined backyard chicken flocks. Single bacterial infection represents 1.82% of the causative agent of respiratory affection, mixed infection of *E. coli* and MG represents 5.5%, while *E. coli* and MS represents 1.82% (Table-5).

In examined commercial chicken flocks with respiratory manifestation, 81.8% flocks were infected with IBV and LPAIV (H9N2). The prevalence of IBV and LPAIV (H9N2) infection was 81.8% and 54.5%, respectively. NDV was not detected in any examined flocks. Mixed viral and bacterial infections were recorded in 63.6% of examined commercial broiler flocks (Table-5).

## Discussion

Respiratory infection with variable clinical manifestations and mortalities increased recently in poultry flocks leading to more economic losses to poultry producers. Respiratory diseases in chickens were usually caused by either single or mixed infections [1,2,22,41-44].

According to our study, the serological prevalence of NDV, IBV, and AIV in backyard chicken flocks was 56.4%, 50.9%, and 12.7%, respectively. Interestingly, 29.1% of the backyard chickens had simultaneous viral infection. Boroomand *et al*. [45] stated that 77%, 45%, and 38.4% of examined birds were serologically positive for NDV, AIV, and IBV, respectively. While Mahzounieh *et al*. [46] found that 85.3% of domestic village chickens in the central part of Iran were seropositive for IBV, while in Mexico the seroprevalence rate of NDV and IBV in backyard village chickens was 2.2% and 56.5%, respectively [47]. In a study, 99% and 18.8% of backyard chickens were seropositive for NDV and AIV, respectively in Grenada [48].

### Viral interaction

The infection of chickens with heterologous viruses mostly results in virus interference or synergism. We found that the common respiratory viruses (IBV, NDV, and low pathogenic avian influenza [LPAI] H9N2) were detected in 50 backyard chicken flocks and nine broiler flocks and, respectively, represent 90.9% and 81.8% of respiratory affections either singly or interacted with other pathogens. The most prevalent respiratory viruses in clinically infected broiler flocks were IBV (81.82%), these results are consistent with previous studies [1-3,22,32,44], demonstrating a high global prevalence of NDV, IBV, and AIV H9N2 either singly or combined other viruses and bacteria.

LPAIV (H9N2) was identified regularly in chicken flocks. Single infection with LPAIV (H9N2) was recorded in one out of 55 examined backyard chicken flocks suffering from decreased feed intake and mild respiratory signs with low MR (6.25%). LPAIV (H9N2) was detected simultaneously with other respiratory pathogens in 12.7% and 54.5% of examined backyard and broiler flocks, respectively, with enhanced pathogenicity leading to more severe clinical out coms with elevated MR, the same results reported by Monne *et al*. [49]. The presence of risk factors such as concurrent viral infections resulted in severe losses of up to 60% mortality. The synergistic effect between IB viruses and H9N2 explained and discussed by Liu *et al*. and Zainab *et al*. [50,51], suggested that the exacerbation of pathogenicity of H9N2 AIV or NDV might be due to enhancing of HA cleavage by trypsin-like serine protease domain, encoded by the open reading frame of the coronavirus vaccine or field strains.

NDV and H9N2 AIV are two of the most economically important viruses that threat poultry production. Simultaneous infection with NDV and H9N2 AIV resulted in MR ranged from 30% to 40% in examined backyard flocks. Other researchers indicated that interaction between NDV and LPAI was reported as they can replicate in the upper respiratory and intestinal epithelial cells by binding to the sialic acid-containing receptors on the cell surface through the hemagglutinin-neuraminidase (HN) or HA protein of NDV or AIV, respectively [52,53]. This pattern of virus replication might be influenced by the previous replication of the other virus in the same site through active antiviral immune responses, including immunomodulatory-induced interferon or recruitment of immune cells [54]. The interaction between pathogens that have the same site of replication might be either synergistic or antagonistic determining the severity of the resulting clinical outcomes. The patterns of interaction can be influenced by the virulence of the strain, time of infection (pre-infection, simultaneously, or superinfection) bird immune response, biological products or metabolites, and/or other environmental risk factors [55,56].

The high prevalence of NDV (46.97%) in unvaccinated backyard flocks resulted from the absence of specific immune response due to lack of vaccination programs, immune suppression induced by other mixed infection such as *E. coli* or other viruses, bacterial infection, stress factors, and/or the absence of biosecurity issues. On the other hand, NDV not detected in examined vaccinated broiler flocks indicated that NDV vaccines are protective and provide good immune response against circulating field NDV strains. Hadipour *et al*. and Munir *et al*. [57,58] reported that previous vaccination with live lentogenic NDV vaccines offered protection for chickens.

### Viral/bacterial interaction

Mixed viral and bacterial infections were respectively detected in 40% and 63.6% of the examined backyard, and broiler flocks with respiratory signs. The most frequently detected mixed infection was IBV and *E. coli* in 23.6% of tested broiler flocks resulting in severe clinical outcomes and an increased MR up to 27.8%. It is clear that the synergistic interaction between respiratory viruses (NDV, H9N2, and IBV) and avian pathogenic *E. coli* and *S. aureus* results in high losses in infected flocks, up to 87.5% in *E. coli* infection and 60% in *S. aureus* infection. Our observation in both backyard and commercial broiler flocks confirms a bidirectional synergistic effect between these concurrently interacting respiratory pathogens in which each pathogen augment pathogenesis of the other one. These bidirectional interactions explain the resulting severe clinical outcomes and higher MR, which coincide with the results of Dadras *et al*. and Mosleh *et al*. [15,30]. *E. coli* infection before, after, or concurrently with LPAIV (H9N2) infection could exacerbate the adverse effects of the LPAIV (H9N2). *E. coli* and LPAIV (H9N2) together can mutually exacerbate the condition of either disease as compared to single infected birds. The synergistic bacterial coinfection occurs by activation of HA cleavage of H9N2 AIV and HN of NDV directly by secretion of trypsin-like protease by protease secreting bacteria [24,59], or indirectly by stimulation of secretion of more proteases by host cells and breakdown of endogenous cell protease inhibitors, activating infection [25] or may be due to the induced immune suppression effect and other stress factors [19,29]. The recorded severe respiratory outcomes and high mortality up to 60% in examined backyard chicken flocks concurrently infected with LPAIV (H9N2) and *S. aureus* may explain these synergistic interactions. The mechanism of *S. aureus* mediated enhancement of LPAIV (H9N2) activation was investigated by Tse and Whittaker [60] who reported that *Staphylococcus* spp. is able to cleave and activate HA by activating plasminogen to plasmin by use of a virulence factor, staphylokinase. Moreover, the high incidence of *E. coli* infection simultaneously with NDV and/or IBV (18/55 of examined backyard flocks) with high mortality and more severe clinical signs increases the hypothesis of the bidirectional synergistic interactions induced by viruses and concurrent bacterial infection. Viral infections induce mechanical damage of ciliated epithelium and goblet cells, which enhance the bacterial adherence and colonization [26,27] or impairment of the phagocytic function and alteration of the innate immune response [28]. Moreover, the synergistic effect due to the interaction between live NDV and IBV vaccines and *E. coli* plays a role in inducing or enhancing colibacillosis in the chicken [29].

Mixed infection of MG and MS with other viruses such as LPAIV (H9N2), IBV was recorded resulting in increased pathogenicity and mortality up to 55.6%. Sid *et al*. and Roussan *et al*. [61,62] reported that clinical symptoms, clinical lesions, and reductions in weight gain were much more significant in mixed infected groups with MG and LPAIV. Concurrent inoculation of chickens with MG has important impacts on the formation of tracheal plugs, increasing pathogenicity of LPAIV (H9N2) [63].

The seroprevalence of MG and MS was 14.5% and 3.6% in backyard flocks, respectively. The concurrent *E. coli* and MG infection-induced more severe respiratory manifestation, including severe airsacculitis, fibrinous pericarditis, and pneumonia, with increased MR, ranged from 1.2% to 20%. It has been reported that challenge with MG and *E. coli* together could induce chronic respiratory disease-like lesions, which indicates that *E. coli* acts synergistically with MG [64].

Our results confirm that mixed infection involving one or more common respiratory viruses, mycoplasma, *S. aureu* s, and avian pathogenic *E. coli* combined with immunosuppressive agents, and unfavorable environmental conditions, are more commonly prevalent and associated with dramatic exacerbations in pathogenicity and mortality. This conclusion was stated by previous studies reporting that multi-infection may have been responsible for high mortalities in poultry flocks [61-64].

## Conclusion

The high prevalence of IBV either as a single or combined infection with LPAIV (H9N2) and/or *E. coli* and in spite of intensive use of commercial vaccines may be due to the failure of the IBV vaccine to protect chickens against field virus infections or circulation of new variant IBV strains. This increases the need for revising vaccination programs as well as strict application of standard biosecurity measures.

The seroprevalence of common respiratory viruses in backyard flocks with no history of pre-immunization with live virus vaccines confirms that exposure to field strains and imposes a great risk that threatens the commercial chicken flocks which in turn acts as a reservoir for most infectious pathogens disseminated to the environment. More efforts should be directed to educate backyard chicken owners to encourage them to implement preventive measures, vaccinate, and apply standard biosecurity issues.

Regular investigation of the currently circulating respiratory infections in both backyard and commercial flocks, as well as the evaluation of vaccination programs, is necessary for the improvement of disease prevention and control.

## Authors’ Contributions

AMA and MHAM contributed to study design, sample collection, viral detection, data analysis, manuscript writing, and reviewing. MMF and AAA shared in isolation and characterization of different bacterial agents and data analysis. TA and IQ shared in data analysis and reviewed the manuscript. All authors read and approved the final manuscript.
